# Cerebral infarcts associated with adenomyosis: a rare risk factor for stroke in middle-aged women: a case series

**DOI:** 10.1186/s12883-018-1213-2

**Published:** 2018-12-19

**Authors:** Xinzhen Yin, Jimin Wu, Shuijiang Song, Baorong Zhang, Yanxing Chen

**Affiliations:** Department of Neurology, the Second Affiliated Hospital, School of Medicine, Zhejiang University, 88 Jiefang Rd, Hangzhou, 310009 China

**Keywords:** Adenomyosis, CA125, Menstruation, Ischemic stroke, Middle-aged women

## Abstract

**Background:**

Adenomyosis is a benign disease with elevated CA125 level.

**Case presentation:**

We report 3 cases with adenomyosis who developed ischemic stroke during menstruation. The levels of CA125, CA19–9, and D-dimer were elevated, which dropped markedly after the menstrual phase. The development of nonbacterial thrombotic endocarditis (NBTE) and stenosis of the cerebral arteries associated with hypercoagulable state and the hyperviscosity nature of the mucinous protein may be the underlying mechanisms.

**Conclusion:**

Our report suggests that adenomyosis might be a risk factor for ischemic stroke in middle-aged patients.

## Background

Adenomyosis is a benign invasion of the endometrium into myometrium, which causes uterine enlargement, dysmenorrheal, menorrhagia, and menorrhalgia [1]. It typically occurs in the third to fifth decade of life. Adenomyosis is not routinely considered as a risk factor for ischemic stroke among young patients (under 50 years of age). Apart from the traditional vascular risk factors, migraine, illicit drug use, patent foramen ovale, oral contraceptives, and pregnancy or puerperium are the most prevalent “rare” risk factors for stroke in young adults [2]. We report here 3 cases with ischemic stroke and adenomyosis, and discuss the necessity to consider adenomyosis as a risk factor for stroke in middle-aged women.

## Case presentation

### Case 1

**Fig. 1:**
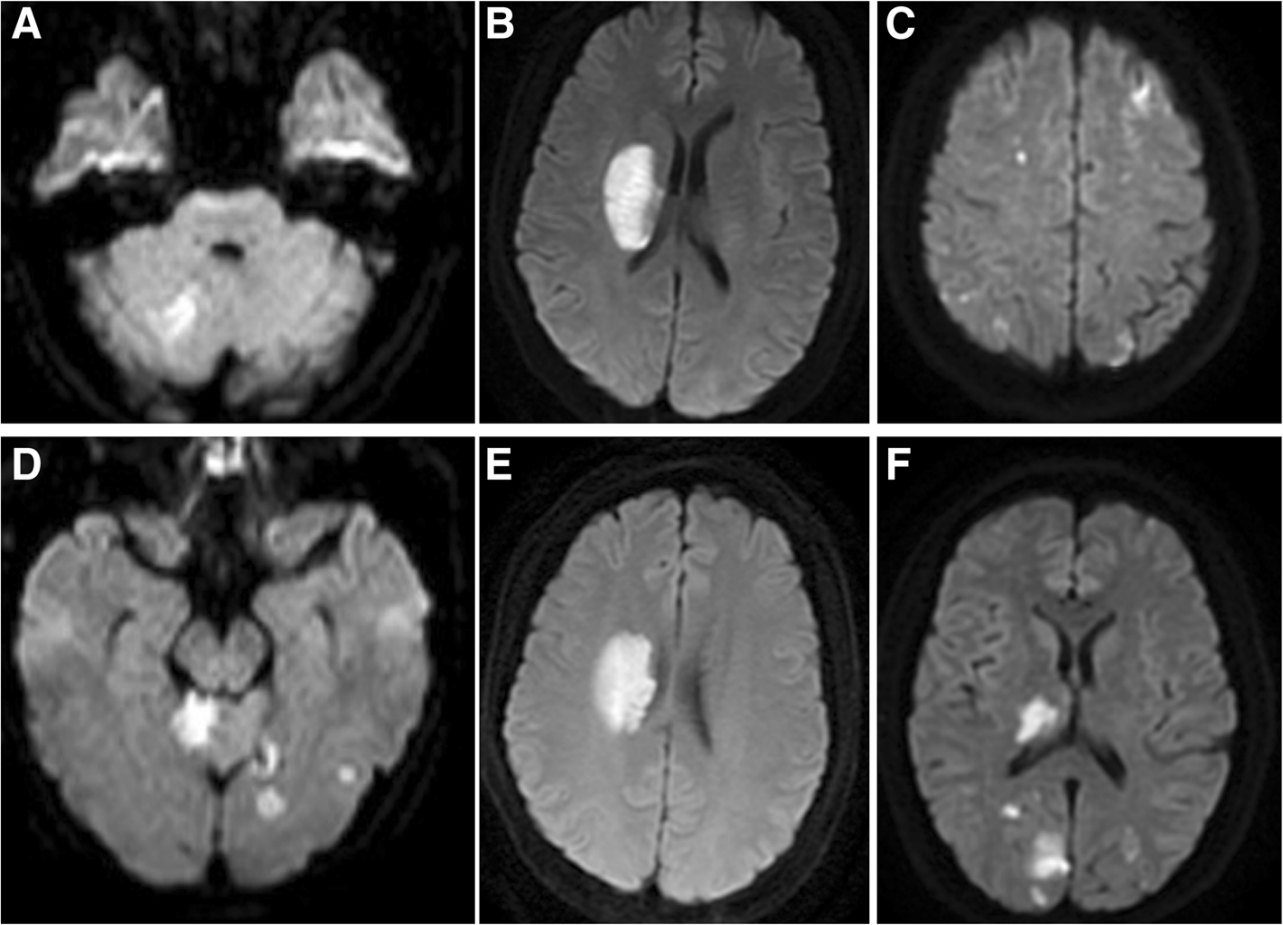
Diffusion weighted imaging findings of the patients. **a**, **d**. Multiple infarctions in the right cerebellum and left temporal lobe (Case 1). **b**, **e**. One infarction in the right basal ganglia (Case 2). **c**, **f**. Multiple infarctions in the right thalamus, occipital lobe, and bilateral frontal and parietal lobes (Case 3)

**Fig. 2:**
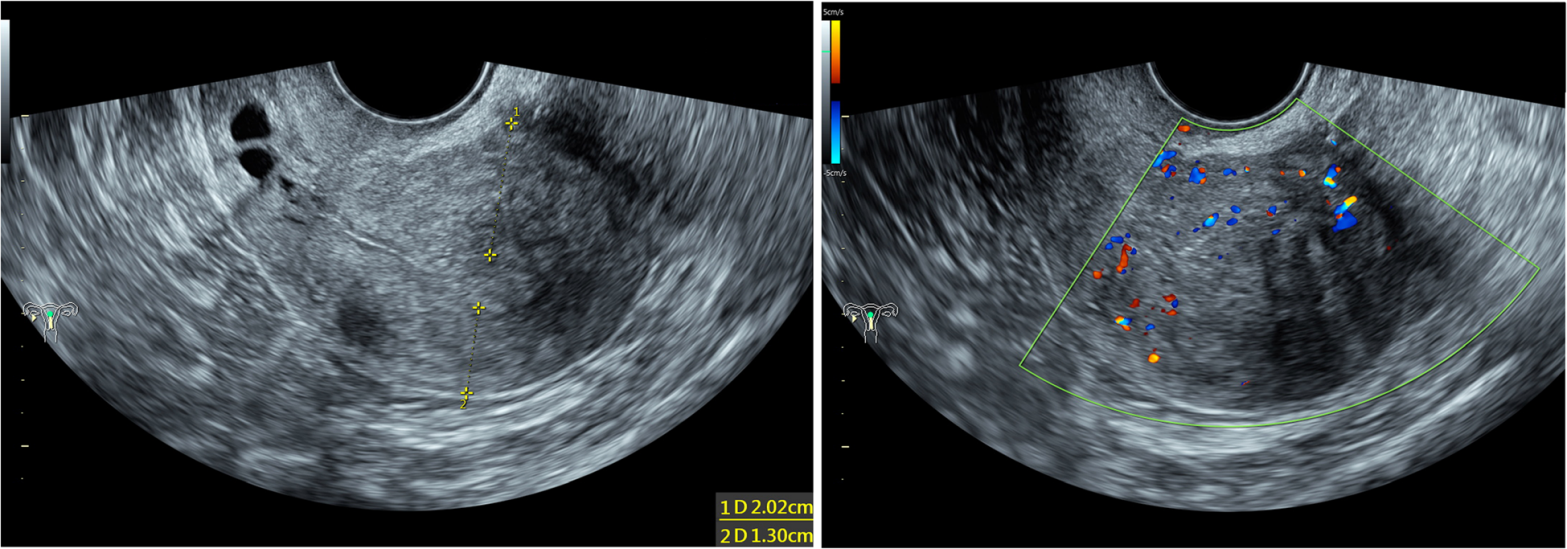
Transvaginal ultrasonography (TVS) of uterus with adenomyosis . TVS of Case 1 shows enlarged uterus, with posterior uterine wall thickening and hypoechoic linear myometrial striations into the myometrium

A 34-year-old woman presented with sudden onset of vertigo and vomiting on the first day of her menstruation. Brain diffusion weighted imaging (DWI) revealed newly occurring multiple infarctions in the right cerebellum and left temporal lobe (Fig. 1). Magnetic resonance angiography (MRA) and carotid CT angiography (CTA) did not show any atherosclerotic changes. Transesophageal echocardiography (TEE) did not reveal any evidence of valvular vegetation. No evidence of arrhythmia was found by ambulatory electrocardiography. Transvaginal ultrasonography (TVS) showed adenomyosis (Fig. 2). Laboratory investigations revealed elevated D-dimer (1050 μg/L; normal range, < 500 μg/L), CA125 (937.1 U/mL; normal range, < 35 U/mL) and CA19–9 levels (462.5 U/mL; normal range, < 37 U/mL). The hemoglobin level was 134 g/L. The D-dimer, CA125 and CA19–9 levels re-evaluated 1 week later were 440 μg/L, 122.9 U/mL and 38.5 U/mL, respectively.

### Case 2

A 37-year-old woman presented with sudden onset of weakness of her left limbs on the second day of her menstruation. DWI revealed newly occurring infarction in the right basal ganglia (Fig. 1). Brain MRA, carotid CTA, TEE, and ambulatory electrocardiography were performed. There was no evidence of arteriosclerosis, cardiac diseases including valvular vegetation and arrhythmia. TVS showed adenomyosis. Laboratory investigations revealed elevated D-dimer (2340 μg/L; normal range, < 500 μg/L), CA125 (735.7 U/mL; normal range, < 35 U/mL) and CA19–9 levels (43.2 U/mL; normal range, < 37 U/mL). The hemoglobin level was 108 g/L. Other laboratory results were normal, including the protein C and protein S activities. Therefore, we re-evaluated the CA125 and CA19–9 levels 1 week later, which were 456.8 U/mL and 50.3 U/mL, respectively.

### Case 3

**Fig. 3:**
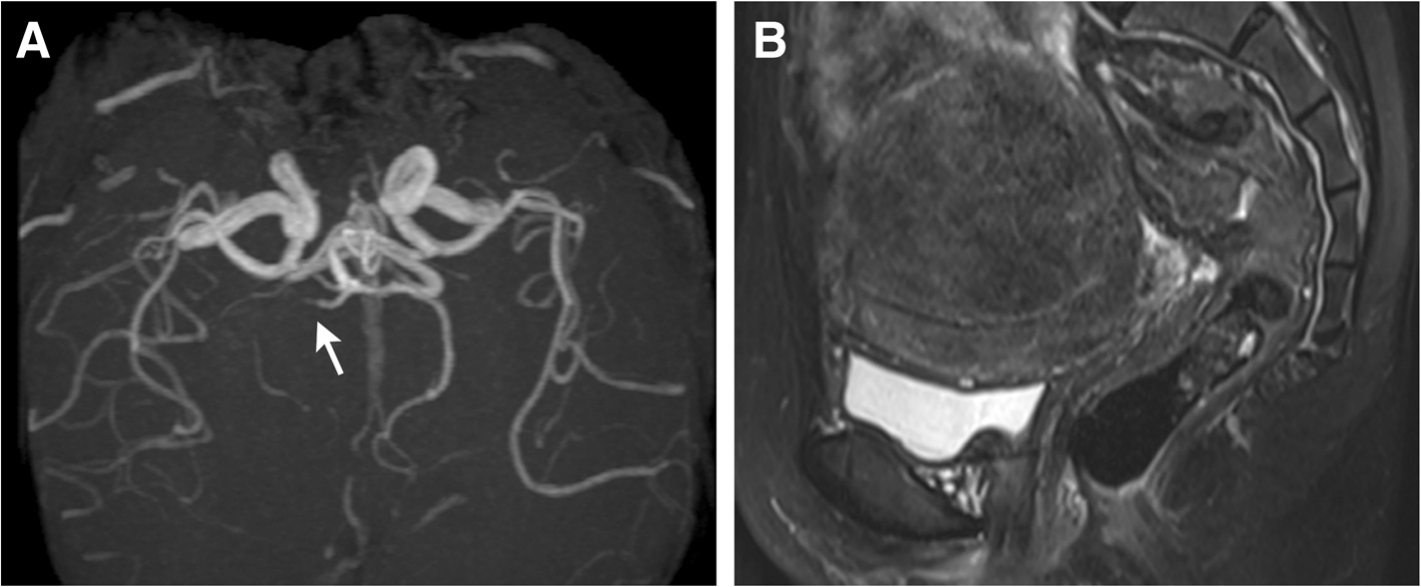
Brain Magnetic resonance angiography (MRA) and pelvic magnetic resonance imaging (MRI) of Case 3. **a**. MRA shows stenosis of the right posterior cerebral artery. **b**. Sagittal T2-weighed MRI shows an enlarged uterus with an ill-defined low signal intensity lesion in the posterior myometrium

A 46-year-old woman developed left hemiplegia on the second day of menstruation. Brain DWI revealed multiple fresh infarcts in the right thalamus, occipital lobe, and bilateral frontal and parietal lobes (Fig. 1). Brain MRA revealed stenosis of the right posterior cerebral artery (PCA) (Fig. 3). The carotid CTA, TEE, and ambulatory electrocardiography findings were normal. Positron emission tomography (PET)/CT showed no malignancies. Pelvic MRI showed an inhomogenenous mass in the uterus (Fig. 3), suggesting of adenomyosis. This was comfirmed by histopathological study when hysterectomy was performed five months later. Laboratory investigations revealed elevated D-dimer (12,040 μg/L; normal range, < 500 μg/L), CA125 (546.5 U/mL; normal range, < 35 U/mL) and CA19–9 levels (1076.6 U/mL; normal range, < 37 U/mL). The hemoglobin level was 121 g/L. The levels of D-dimer, CA19–9, and CA125 re-evaluated 1 week later were 2200 μg/L, 213.7 U/mL, and 193.9 U/mL, respectively. After hysterectomy, the levels of D-dimer, CA19–9, and CA125 returned to within normal ranges, and no infarction recurred.

## Discussion and conclusion

We report in this study 3 cases who developed ischemic stroke during their menstruation. They did not have any cerebrovascular risk factors. Elevated CA125, CA19–9, and D-dimer levels were observed, which dropped significantly during the non-menstrual phase. All of them have adenomyosis. The levels of these markers returned to within normal ranges following hysterectomy in one of the patients.

In all the 3 patients, both the levels CA125 and CA19–9 were elevated, especially the CA125 level. However, no malignancies were found. CA125 is a member of the mucin family glycoproteins. Elevate serum CA125 level is most commonly seen in women with epithelial ovarian tumors, but also with endometriosis, pelvic inflammatory disease or adenomyosis [3]. Cerebral infarcts in adenomyosis patients have been previously reported before mostly in Japan. We summarized the characteristics of all the12 cases (including the current 3 cases) in Table 1. It is suggested that increased CA125 levels might play a role in the hypercoagulable state of the patients, which leads to the development of NBTE and increased aggregation of white and red blood cells [4]. Patients with adenomyosis are at risk of having an activated coagulation system, which leads to increased risk of thrombotic disorders [5]. Systemic embolism in the fingers or kidneys, as well as thrombi in the brachiocephalic trunk and left subclavian artery have been reported in adenomyosis cases [6]. NBTE, detected by TEE, has been found as the embolic source in 3 ischemic stroke patients with adenomyosis (Table 1). These reports indicate an underlying thromboembolic mechanism in adenomyosis patients with ischemic stroke. Although we did not confirm the existence of NBTE in our patients, a mechanism of thromboembolism can not be ruled out. Most NBTEs are discovered on autopsy but not antemortem, which may result from the difficulty in detecting these small sized vegetations under the cardiac valve [7]. It is also possible that the vegetations have already detached from the cardiac valve at the time of evaluation following the ischemic events.

**Table 1:** Cases with adenomyosis associated stroke

Reference	Age	Occurance during menstruation	CA125 (U/mL, normal < 35)	CA19–9 (U/mL, normal < 37)	NBTE	D-dimer^b^ (μg/L)
1 [6]	42	Yes	1750	/	No	6000
2 [6]	45	No	159	/	No	1100
3 [6]	44	No	/	/	No	/
4 [6]	50	Yes	42.6	/	No	570
5 [10]	59	/	334.8	/	Yes	7000
6 [7]	49	No	379	69.2	Yes	3990
7 [11]	48	/	901	1791	Yes	1900
8 [12]	42	/	395	/	No	1400
9 [12]	50	/	143	/	No	3700
10^a^	34	Yes	937.1	462.5	No	1050
11^a^	37	Yes	735.7	43.2	No	12,040
12^a^	46	Yes	546.5	1076.6	No	2340

^a^Present cases; ^b^normal, <1000μg/L for the reported cases; slash indicates not mentioned

Apart from thromboembolism, we believe stenosis of the cerebral artery may also be one of the mechanisms underlying adenomyosis associated ischemic stroke. Brain MRI revealed stenosis of the right PCA and ischemia in its territory in the third patient. Because of its hyperviscosity nature, CA125 has been suggested to be associated with the stenosis or occlusion of cerebral arteries by this mucinous protein itself [4]. However, further studies are needed to confirm this hypothesis.

All the 3 patients developed ischemic stroke in the menstrual phase. Consistently, another 2 cases were also confirmed to be in menstruation when they initially developed symptoms (Table 1). The serum CA125 levels vary at the different phases of the menstrual cycle, which peak during the menstruation [8]. The menstrual CA125 level can exceed the normal limit even in healthy women. The elevation of serum CA125 during menstruation is thought to be related with endometrial cell surface antigen shed into the systemic circulation or peritoneal irritation. In the current study, marked increased CA125 and D-dimer levels were detected during menstruation, indicating activated coagulation system associated with CA125. Besides, menstruation induced activation of the tissue factor coagulation pathway may also play a role in the abnormal coagulation of the adenomyosis patients [9]. Therefore, patients with adenomyosis are more likely to develop cerebral infarction during menstruation.

In conclusion, we report 3 adenomyosis patients who developed ischemic stroke during menstruation. These patients may be at risk of hypercoagulability associated with increased CA125 level and menstruation-related activation of coagulation pathway. The development of NBTE and stenosis of the cerebral arteries may be the underlying mechanisms leading to the cerebral infarction. Therefore, it is important to be aware of the adenomyosis as a risk factor for ischemic stroke in middle-aged patients. To our knowledge, this is the first report of Chinese cases with adenomyosis associated ischemic stroke.

## Abbreviations

CTA: Carotid CT angiography
DWI: Diffusion weighted imaging
MRA: Magnetic resonance angiography
NBTE: Nonbacterial thrombotic endocarditis
PCA: Posterior cerebral artery
PET: Positron emission tomography
TEE: Transesophageal echocardiography
TVS: Transvaginal ultrasonography
